# Browning of White Adipose Tissue Uncouples Glucose Uptake from Insulin Signaling

**DOI:** 10.1371/journal.pone.0110428

**Published:** 2014-10-14

**Authors:** Karin Mössenböck, Alexandros Vegiopoulos, Adam J. Rose, Tjeerd P. Sijmonsma, Stephan Herzig, Tobias Schafmeier

**Affiliations:** 1 Joint Division Molecular Metabolic Control, DKFZ-ZMBH Alliance and Network Aging Research, German Cancer Research Center (DKFZ) Heidelberg, Center for Molecular Biology (ZMBH) and University Hospital, Heidelberg University, Heidelberg, Germany; 2 Junior research group Metabolism and Stem Cell Plasticity, German Cancer Research Center (DKFZ), Heidelberg, Germany; Tohoku University, Japan

## Abstract

Presence of thermogenically active adipose tissue in adult humans has been inversely associated with obesity and type 2 diabetes. While it had been shown that insulin is crucial for the development of classical brown fat, its role in development and function of inducible brown-in-white (brite) adipose tissue is less clear. Here we show that insulin deficiency impaired differentiation of brite adipocytes. However, adrenergic stimulation almost fully induced the thermogenic program under these settings. Although brite differentiation of adipocytes as well as browning of white adipose tissue entailed substantially elevated glucose uptake by adipose tissue, the capacity of insulin to stimulate glucose uptake surprisingly was not higher in the brite state. Notably, in line with the insulin-independent stimulation of glucose uptake, our data revealed that brite recruitment results in induction of solute carrier family 2 (GLUT-1) expression in adipocytes and inguinal WAT. These results for the first time demonstrate that insulin signaling is neither essential for brite recruitment, nor is it improved in cells or tissues upon browning.

## Introduction

According to estimations of the WHO, nowadays more than 10% of the world’s adult population is obese. Obesity frequently entails detrimental secondary diseases and therefore represents the fifth leading risk for global deaths [1]. In contrast to the energy storing function of white adipose tissue (WAT), brown adipose tissue (BAT) has been long known in small mammals and newborns to defend body temperature at the expense of increased energy consumption through thermogenesis by uncoupling the electron transport chain from ATP synthesis via uncoupling protein-1 (UCP-1) [2]. Research on BAT has recently been revived by PET/CT-studies demonstrating presence of metabolically active BAT in discrete areas of adult humans, [3]–[5]. Importantly, active BAT has the potential to enhance systemic glucose disposal and improve global insulin sensitivity in human subjects [6], [7]. Thus, increasing BAT activity is considered a promising option for innovative weight lowering therapies and amelioration of hyperglycemia and insulin resistance, which are hallmarks of type 2 diabetes and the metabolic syndrome, respectively [8].

In addition to classical BAT, a novel type of inducible “brown-like” adipose tissue has been discovered some years ago. This UCP-1 expressing tissue is referred to as beige, or brite (brown-in-white) adipose tissue and has been shown to develop within WAT depots upon cold or β-adrenergic stimulation [9]–[12]. Brite adipocytes display multilocular lipid droplets, comparable levels of UCP-1 protein and similar physiological functions as classical brown adipocytes. Nonetheless, they are clearly separate cell types, as they derive from distinct progenitors and display specific gene expression signatures [9], [13]–[15]. It is not entirely clear if the BAT detected in humans consists of classical brown or brite adipocytes or a mixture [14], [16]–[19]. It is still under debate if the interconversion of WAT to brite adipose tissue is caused by de-novo differentiation of precursor cells, by trans-differentiation of existing white adipocytes, or both [20], [21]. Nevertheless, the inducibility of brite adipocytes by pharmacological and nutritional cues renders them an attractive target for increasing thermogenesis in obese and/or diabetic patients [9], [22].

Insulin and insulin-like growth factor (IGF)-1 have been shown to regulate gene expression in preadipocytes and are therefore central for coordination of adipocyte differentiation [23], [24]. A recent study investigating mice lacking both insulin and IGF-1 receptors in adipose tissues revealed that WAT and BAT mass were markedly reduced in these animals, thereby confirming a critical role of insulin/IGF-1 signaling in adipose tissue development in vivo [25]. Brown adipocyte differentiation as well as basal BAT activity was substantially compromised upon ablation of both receptors. Surprisingly, knockout mice displayed an advantageous metabolic phenotype and increased energy expenditure, and the residual BAT still responded to β_3_-adrenergic stimulation [25].

These studies however did not investigate the role of insulin in the process of browning of WAT and in the function of brite adipose tissue, which prompted us to address function of insulin in a brite adipose tissue context. Disturbances of the insulin signaling pathway might affect brite adipocyte recruitment and/or activity, which could aggravate insulin resistance in a type 2 diabetic setting. Vice versa, browning of adipose depots might increase their insulin-sensitivity, thereby systemically improving the metabolic state. The results of our study indicate a key role of insulin signaling in white and brite adipocyte differentiation. However, our data demonstrate that WAT does not require insulin for browning and - more important - that brite adipose tissue, despite displaying enhanced glucose uptake capacity, is not more sensitive to insulin signaling compared to WAT.

## Materials and Methods

### Progenitor isolation and cell culture

Media and supplements were purchased from Invitrogen unless stated otherwise. All antibodies were obtained from eBioscience. DMEM with 4.5 g/l glucose was used unless stated otherwise. Primary stromal-vascular fraction (SVF) derived Lin^−^Sca1^+^ adipocyte progenitor cells [26] were isolated from inguinal white adipose tissue of male C57Bl6 mice (Charles River) at age 6–8 weeks. Progenitor isolation has been approved by the DKFZ ethical committee (DKFZ252) and was performed previously in our lab [27]. Tissues were minced, digested with 1.5 mg/ml collagenase type II (Sigma) in DMEM (4.5 g/l glucose) supplemented with 0.5% bovine serum albumin (BSA) (Sigma), 15 mM HEPES, 3.2 mM CaCl_2_, 10% fetal bovine serum (FBS) and 0.05 mg/ml DNase I (Roche) for 30 to 50 min shaking at 37°C. The cell suspension was strained through a 300 µM nylon mesh (Neolab), collected by centrifugation, washed in BSA-buffer (1x PBS supplemented with 0.5% BSA and 1 mM EDTA) and strained again through a 70 µM cell strainer (BD biosciences). Subsequently cells were incubated on ice for 10 min with anti-CD16/32, then for 30 min additionally with Lin (anti-CD31-biotin, anti-CD45-biotin and anti-Ter119-biotin), washed twice with BSA buffer and incubated on ice for 15 min with streptavidin microbeads (Miltenyi Biotech). After washing with BSA-buffer, cells were separated using MS columns and the Octo MACS Separator Starter Kit (Miltenyi Biotech), collecting the Lin^−^ cell fraction by centrifugation of the flow. Those cells were incubated on ice for 30 min with anti-Sca1-PE-Cy7, washed and bound to Anti-Cy7 microbeads (Miltenyi Biotech) for 15 min. Then cells were subjected to the separation columns again, discarding the flow through and eluting the Lin^−^Sca1^+^ fraction by removal of the magnet. Cells were counted, seeded at 10^4^ cells/well and cultivated to confluency in laminin (Santa Cruz) coated cell culture plates in DMEM, 10% FBS, 1% penicillin/streptomycin (pen/strep), and 10 ng/ml murine bFGF (R&D Systems). Differentiation was induced by exchanging the medium to DMEM, 10% FBS, 1% pen/strep, 500 nM dexamethasone (Sigma), 1 µg/ml insulin (Sigma), 3 nM triiodothyronine (T3) (Sigma), and for brite differentiation 1 µM carbaprostacyclin (cPGI_2_) (BIOZOL) or ethanol as vehicle control for white differentiation (day0). After 2 days the medium was replaced by DMEM, 5% FBS, 1 µg/ml insulin, 3 nM T3 and 1 µM cPGI_2_ or ethanol. The medium was exchanged every day. Cells were harvested on day 8, 3 h before harvesting the medium was replaced by DMEM with or without 1 µM norepinephrine (NE) (Sigma).

### siRNA mediated gene knockdown

Primary iWAT derived stromal-vascular fraction (SVF) derived Lin^−^Sca1^+^ adipocyte progenitor cells were isolated and plated as described above and transfected with 50 nM siRNA 2 days prior to induction of differentiation, using Mm_Slc2a1_5 FlexiTube siRNA or Allstars Negative Control siRNA (Qiagen). Nucleic acids were pre-incubated with DharmaFECT 1 siRNA Transfection Reagent (Dharmacon) for 20 min before adding them to the cells.

### GLUT-1 inhibition

Primary iWAT derived stromal-vascular fraction (SVF) derived Lin^−^Sca1^+^ adipocyte progenitor cells were isolated and plated and differentiated as described above. For the last 2 days the glucose transporter inhibitor III STF-31 (Calbiochem) was added to the medium at a concentration of 1 µM according to previous reports [28].

### RNA isolation and quantitative RT-PCR

Cells and tissues were harvested in QIAzol (QIAGEN), tissues were additionally lyzed in a tissue lyzer (QIAGEN). RNA was isolated using the RNeasy Micro- and -Mini Kit (QIAGEN) for cells and tissues, respectively, performing on-column DNAse digest. 100 to 1000 ng RNA were reverse transcribed with the SuperScript II Kit (Invitrogen) and Oligo(dT) primers (Fermentas), then cDNA was diluted to a final concentration of 1 ng input RNA per µl cDNA and 5 µl were used for RT-PCR. Detection of gene expression was performed using the Taqman system with Taqman Gene Expression Master Mix and specific Gene Expression Assays and an ABI StepOnePlus sequence detector (Applied Biosystems). mRNA levels relative to TBP expression were calculated by the delta Ct method.

### Microarray analysis

Lin^−^Sca1^+^ adipocyte progenitor cells were isolated and cultured to confluency as described above. Then adipogenic differentiation with 1 µM cPGI_2_ or ethanol was induced with or without the presence of 1 µg/ml insulin. After 24 h cells were harvested, RNA was isolated as described and subjected to gene expression analysis using mouse430_2 arrays (Affymetrix), the group size was 3 arrays per condition. cRNA was synthesized and hybridized according to the manufacturer’s recommendations. Annotation of the arrays was performed with a CustomCDF (Version 14) with Entrez based gene definitions. The Raw fluorescence intensity values were normalized by quantile normalization. Differential gene expression was analyzed based on loglinear mixed model ANOVA [29], [30] with a commercial software package (SAS JMP7 Genomics, version 4) (SAS Institute, Cary, NC, USA). Significance was assumed based on a false positive rate of p = 0.05 with FDR correction.

### Protein analysis

Cells were harvested in protein lysis buffer consisting of 25 mM Tris-HCl (pH 7.4), 100 mM NaCl, 1 mM EDTA, 0.5% Triton-X100, 0.5% NP-40, 1x proteinase inhibitor cocktail (PIC) (Sigma), 0.5 mM Na_3_VO_4_, 10 mM NaF and 10 mM C_3_H_7_Na_2_O_6_P. Protein concentration was quantified using the BCA Protein Assay (Pierce) and 20 µg of protein were loaded to 10% SDS-polyacrylamide gels and blotted to nitrocellulose membranes. The antibodies used for Western Blot were specific for thymoma viral proto-oncogene 1 (AKT), p-AKT (9272 and 9271, Cell Signaling), GLUT-1 (abcam ab32551), and VCP (ab11433, Abcam) for normalizing. Quantification was performed using the ImageJ software.

### Animals

Male C57Bl6 mice were purchased from Charles River Laboratories (CRL) at age 6–8 weeks. For the ablation of pancreatic β-cells 13-week old mice were i.p. injected daily with 60 µg/g body weight streptozotocin (STZ) (Axxora) or 0.05 M sodium citrate (Sigma) as vehicle control for 6 consecutive days. Random fed and 16 h fasted blood glucose levels were determined 3 weeks later using an automatic glucose monitor (One Touch, Lifescan) and compared to random fed blood glucose levels before the STZ injections to verify the diabetic state. For diet studies 8-week old mice were fed with low fat diet (LFD) (10% calories from fat) or high fat diet (HFD) (60% calories from fat) (Research Diets) for 12 weeks. Total body fat mass was determined by magnetic resonance imaging on an EchoMRI-100 quantitative NMR body composition analyzer (Echo Medical Systems). Organs and serum were collected, washed in PBS, snap-frozen in liquid nitrogen and stored at −80°C. aWAT (intra-abdominal visceral WAT) was dissected from the abdominal cavity, iWAT (equivalent to subcutaneous WAT) was dissected from the layer under the skin and outside of the abdominal cavity at the hips. BAT was dissected from the interscapular region. All lymph nodes, as well as adjacent WAT in BAT, were removed prior to further processing. For cold exposure, female NMRI mice were purchased from Charles River Laboratories (CRL) at age 8–10 weeks and housed at 23°C and 5°C respectively for 10 days. All other mice were housed at ambient temperature (24°C) on a 12 h light-dark cycle.

### Ethics statement

All animal handling procedures were performed in accordance with the European Union directives and the German animal welfare act and have been approved by local authorities (Regierungspräsidium Karlsruhe, Az. 35-9185.81/G-82/12).

Adipocyte progenitor isolation has been approved by the DKFZ ethical committee (DKFZ252).

### Osmotic pumps

For β-adrenergic stimulation to induce browning of white adipose tissue (WAT), CL316243 (CL) (Tocris Bioscience) in 0.9% NaCl or vehicle was administered at continuous rates at a dose of 1 µg/g BW/day by subcutaneous implantation of an osmotic pump (ALZET model 1002). After 10 days mice were sacrificed.

### In vitro glucose uptake assay

Lin^−^Sca1^+^ adipocyte progenitor cells were isolated and differentiated into white and brite adipocytes respectively as described above. 24 h before the assay the medium was replaced with DMEM (1 g/l glucose) supplemented with 0.5% BSA (Sigma) ± cPGI2 and ± STF-31. The uptake essay was performed according to established protocols [31].

### ITT and in vivo glucose uptake assay

8-week old male C57Bl6 mice were implanted with osmotic pumps containing CL or vehicle and body fat mass was determined by magnetic resonance imaging as described above. 10 days later, on the day of the experiment, the initial blood glucose value was determined, then animals were i.p. injected with 240 µCi/kg body weight ^3^H-2-deoxyglucose (^3^H-2-DOG) and with 0.5 U/kg body weight Huminsulin 100 (Lilly) or vehicle, all in 0.1 mM 2-DOG. Every 15 min, blood glucose was determined and 25 µl of blood were collected. After 45 min the animals were sacrificed and organs and serum were collected, washed in PBS, snap-frozen in liquid nitrogen and stored at −80°C. For analysis tissue weights were recorded and tissue was homogenized with a Mikro-Dismembrator S (Sartorius) in 1 ml tissue lysis buffer consisting of 50 mM Tris, 1 mM EDTA, 10 mM NaF, 2 mM Na_3_VO_4_, 1 mM DTT, 150 mM NaCl and 1% NP40, pH 7.5. Lysates were centrifuged at 4000 g for 20 min at 4°C and part of this supernatant (S1) was supplemented with 0.1 M Ba(OH)_2_ and 0.1 M ZnSO_4_ (Sigma) to derivatize and precipitate phosphorylated ^3^H-2-DOG. Following a centrifugation 4000 g for 20 min at 4°C this supernatant (S2) was saved for determination of not taken up (underivatised) tracer. S1 and S2 were subjected to scintillation counting as described above. Also blood was supplemented with 0.1 M Ba(OH)_2_ and 0.1 M ZnSO_4_, centrifuged and the supernatant was used for scintillation counting. For SAM (as described) the injected insulin/^3^H-2-DOG/2-DOG solution was used. For a detailed description of calculation procedure, please note supplementary information.

### Calculation of glucose-uptake

Tissue-specific glucose uptake rate was calculated by using average plasma ^3^H activity, calculated as area under the curve, and average plasma glucose concentration as described [32] with modifcations as described elsewhere [33], [34].

### Immunohistochemistry

Tissues were subjected to 24 h fixation in 4% paraformaldehyde, washed for 2 h in running tap water and stored in 70% ethanol for up to several weeks until paraffine-embedding, then tissues were sliced and dried. For staining slices were stepwise de-paraffinated, boiled in 10 mM citrate buffer for antigen retrieval, blocked for 1 h with 1% BSA and stained with 1∶50 anti-Ucp1 (ab23841, Abcam) antibody over night at 4°C. After incubation with secondary antibody (172–1019, BioRad), slides were subjected to 3,3′-diaminobenzidine (DAB) (Sigma) staining and hematoxylin (Sigma) – eosin (Roth) counterstaining, then mounted (Dako) and examined by light microscopy (Zeiss).

### Statistical analysis

The results are shown as means ± SEM. Statistical analysis was performed by two-tailed student’s t test, For RT-PCR data this was calculated on log10-transformed data, significance was assumed at p<0.05. For pairwise multiple comparison procedures an ANOVA analysis based on the Student-Newman-Keuls Method was performed.

## Results

### Lack of insulin does not impair browning capacity of primary pre-adipocytes

**Figure 1:**
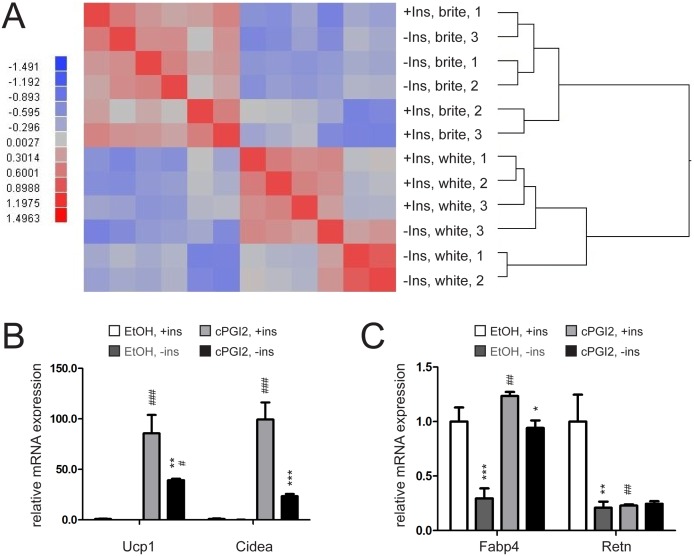
Lack of insulin impairs differentiation but not browning capacity of primary pre-adipocytes. (**A**) Heatmap showing differential mRNA expression between confluent primary inguinal white adipose tissue (iWAT) precursor cells differentiated for 24 h with white (EtOH treated) or brite (cPGI_2_ treated) differentiation cocktail and between absence or presence of insulin (Ins) in the medium. Higher and lower expression is displayed in red and blue, respectively. (n = 3). (**B**) mRNA expression of UCP-1 and CIDEA or (**C**) FABP4 and RETN in primary iWAT precursor cells differentiated into white (EtOH treated) or brite (cPGI_2_ treated) adipocytes for 8 days with insulin present in the differentiation medium for the indicated timepoints (n = 3). All values in bar graphs are expressed as means ± SEM, #p<0.05, ##p<0.01, ###p<0.001 white (EtOH treated) vs. brite (cPGI_2_ treated) cells, *p<0.05, **p<0.01, ***p<0.001 normal conditions vs. insulin deprived conditions.

The previous observation that insulin signaling is crucial for proper brown adipocyte differentiation and adipose tissue development [25], [35], prompted us to screen for insulin-dependent gene expression patterns in white and brite adipocytes during differentiation. To this end, we isolated primary adipocyte precursor cells from inguinal fat depots of C57Bl6 mice. Browning of adipocyte precursor cells was induced by prostacyclin (cPGI_2_)-treatment which has been described previously to act downstream of β-adrenergic stimulation thereby shifting adipocyte progenitors to a brite phenotype [36]. Cells were incubated in the absence or presence of insulin in the medium for 24 h and subsequently harvested. Insulin response of the progenitor cells was verified by Western blot analysis using a phospho-AKT-specific antibody (Fig. S1A, B). RNA was isolated and gene expression was analyzed by Affymetrix microarrays. As expected, bioinformatic exploration of microarray data revealed distinct expression profiles of preadipocytes differentiated in absence or presence of cPGI_2_ in a hierarchical clustering analysis on correlations. However, there was little impact of insulin on the gene expression levels of both white and brite adipocytes in the correlation heat map, indicating that insulin has no further effects on global gene expression in the initial phase of adipogenic differentiation (Fig. 1A). We next differentiated adipocyte progenitors for 8 days into white and brite adipocytes with insulin present and absent in the medium. Expression of selected marker genes was determined in order to assess the role of insulin availability during adipogenic differentiation. The adipogenic program was activated largely independent of the presence of insulin in the medium, as assessed by expression of the early adipogenic marker gene PPARγ [37] and its co-factor PGC1α (Fig. S1C). In addition, the expression of the brite markers UCP-1 and cell death-inducing DNA fragmentation factor, alpha subunit-like effector A (CIDEA) was only marginally affected by insulin in the basal, white state (without cPGI_2_, Fig. 1B). Notably, the profound induction of these brite markers upon stimulation by chronic cPGI2 treatment was markedly decreased if insulin was not present in the medium (Fig. 1B). Furthermore, despite unaltered early adipogenic differentiation markers, absence of insulin reduced the overall efficacy of differentiation as assessed by the expression of the later “white” adipogenic marker resistin (RETN) [38] and the mature adipocyte marker fatty acid binding protein [39] (FABP4, Fig. 1C). FABP4 was upregulated by chronic cPGI2 treatment regardless the presence or absence of insulin. However, cells differentiated without insulin still exhibited a significantly lower FABP4 expression (Fig. 1C). Altogether, our data show that insulin is crucial for regular adipocyte differentiation while it does not arrest the browning process.

### Browning of white adipose tissue is functional in diabetes mouse models

**Figure 2:**
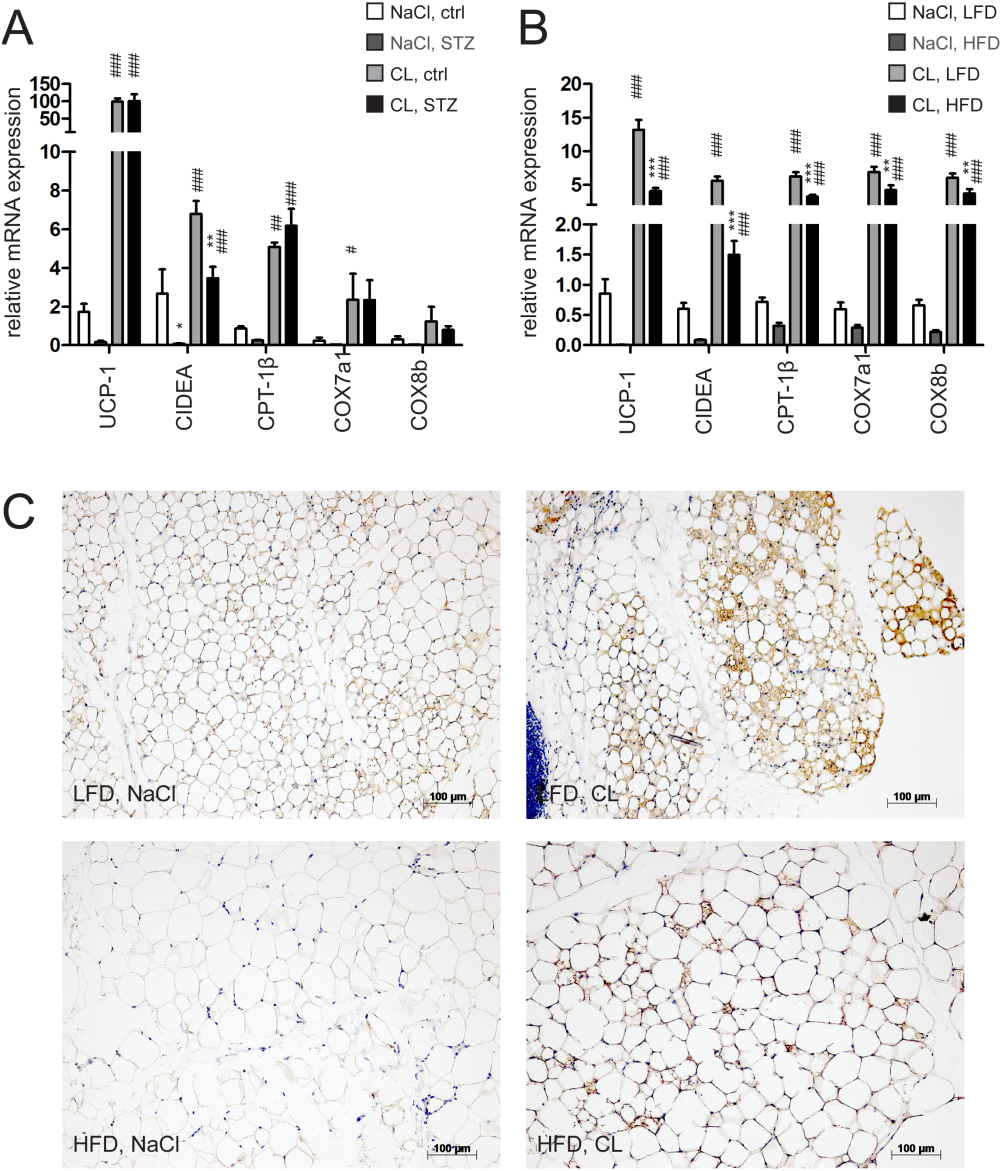
Defective insulin signaling impairs basal brite state while still allowing efficient brite recruitment in WAT. (**A**) mRNA expression of UCP-1, CIDEA, CPT-1β, COX7a1, COX8b in inguinal white adipose tissue (iWAT) of mice injected with streptozotocin (STZ, 60 µg/day/g bw) and control mice or (**B**) of 12 weeks low fat diet (LFD) or high fat diet (HFD) fed mice implanted with CL316,243 (CL, dose of 1 µg/g/day) or control (NaCl) loaded s.c. osmotic pumps for 10 days. (**C**) UCP-1 stained iWAT slices of mice fed LFD or HFD for 12 weeks implanted with NaCl or CL s.c. pumps. All values in bar graphs are expressed as means ± SEM, n = 7–10, #p<0.05, ##p<0.01, ###p<0.001 NaCl vs. CL, *p<0.05, **p<0.01, ***p<0.001 control vs. STZ and LFD vs. HFD treated animals, respectively.

To test the impact of insulin on browning in vivo, we employed established mouse models of insulin deficiency (streptozotocin (STZ) induced β-cell ablation) and insulin resistance (high fat diet (HFD) induced obesity). Serum glucose and insulin levels were determined in these mice in order to verify pathological settings (Fig. S2A, B). Metabolic dysfunction in these animals was further confirmed by demonstration of weight/adipose tissue loss in the STZ and gain of weight/adiposity in the HFD model (Fig. S2C, D). In both mouse models, we subsequently induced white adipose tissue browning by application of the β_3_-adrenoreceptor-specific sympathomimetic CL316243 (CL) through subcutaneously implanted osmotic pumps. Browning efficiency with this method was equal as compared to repeated i.p. injections as indicated by marker gene expression and reduction in adipose tissue mass after 10 days treatment (Fig. S2E, F). In accordance, CL treatment resulted in a reduction of body fat mass without affecting BAT, liver and skeletal muscle mass in control animals. Adipose tissue mass was virtually unaffected by chronic CL administration in STZ-treated animals (Fig. S2G–H) which can be explained by substantial loss of fat mass prior to CL-treatment caused by the insulin deficiency (Fig. S2D). We then quantified expression of brite markers in inguinal (iWAT, equivalent to subcutaneous WAT) and abdominal white adipose tissue (aWAT, intra-abdominal visceral WAT) depots of diabetic and non-diabetic animals. The basal expression of UCP-1, CIDEA and carnitine palmitoyltransferase 1b (CPT1b) was significantly reduced in iWAT and aWAT upon β-cell ablation (Fig. 2A, Fig. S2I, upper panel). However, after CL treatment, brite marker transcript levels of diabetic mice increased to the levels of controls (Fig. 2A). Given the markedly lowered basal expression, the fold change of brite marker mRNAs was even higher in STZ-treated mice.

In the high fat diet (HFD) fed animals exhibiting hyperinsulinemia and insulin resistance, CL treatment resulted in decreased body fat mass and reduced serum insulin as it did in control (LFD fed) animals (Fig. S2B and G lower panel). The basal expression of brite markers in iWAT depots was compromised in a similar way as in the STZ model (Fig. 2B). However, here the marker expression reached lower end levels after CL treatment compared to insulin deficient mice. Notably, the relative increase compared to control (fold change) animals was still higher in HFD than in LFD fed mice (Fig. 2B). The situation in aWAT was similar, although brite markers were generally expressed at low levels (Fig. S2I lower panel). In summary, gene expression data suggest that ablated insulin signaling affected the basal expression of brite markers in WAT but did not compromise browning capacity.

To confirm these results, we performed immunohistochemical analysis of UCP-1 in inguinal and abdominal adipose tissue depots from normal and HFD fed mice. We found absolute levels of UCP-1 to be reduced in HFD fed animals under both control and CL conditions. The relative increase of UCP-1 protein levels however was substantial also in HFD fed animals, thereby qualitatively reflecting mRNA expression data (Fig. 2C, Fig. S2J). Altogether, these findings indicate that while unstimulated WAT depots display lower brite marker expression as a result of aberrant insulin signaling status, β_3_-adrenergic stimulation still could efficiently induce the browning program. Even if a slightly compromised brite endstate apparently was achieved in the disease models, it is still obvious that recruitment of UCP-1 positive adipocytes within WAT depots can efficiently take place independently of insulin signaling.

### Browning of white adipose tissue increases systemic glucose clearance in an insulin-independent manner

**Figure 3:**
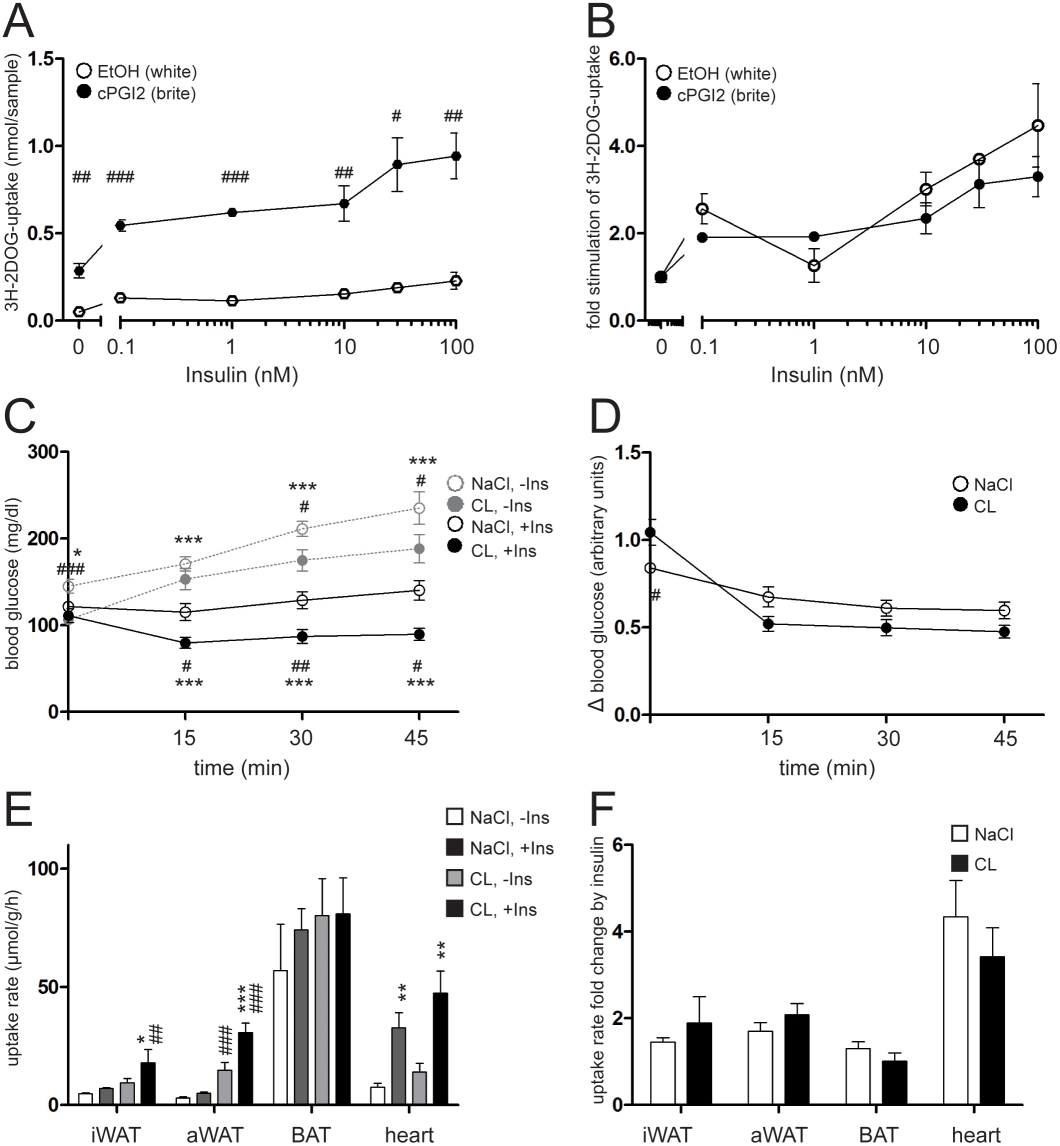
Brite adipose cells and tissues exhibit elevated glucose uptake independent of insulin stimulation, thereby enhancing glucose clearance from blood. (**A, B**) ^3^H-2-deoxy-D-glucose (3H-2DOG) uptake by primary inguinal white adipose tissue (iWAT) precursor cells (**A**) absolute or (**B**) relative to unstimulated basal uptake. Cells were differentiated into white (EtOH treated) or brite (cPGI_2_ treated) adipocytes for 8 days and stimulated with different doses of Insulin for 20 minutes. (**C, D**) Intraperitoneal insulin (Ins) tolerance test (0.5 U/kg body weight insulin) of mice treated with CL316,243 (CL, 1 µg/g/day) or NaCl via s.c. implanted osmotic pumps for 10 days. (**C**) Absolute blood glucose levels and (**D**) levels relative to non-insulin-stimulated are shown. (**E, F**) 3H-2DOG uptake rate into inguinal or abdominal white (iWAT, aWAT) or brown (BAT) adipose tissue and heart of the same mice as in B, C. (**E**) Absolute uptake and (**F**) uptake relative to non-insulin-stimulated conditions is shown. Uptake rates were measured 45 minutes after intraperitoneal injection of insulin or vehicle. All values are expressed as means ± SEM, n = 3–6, #p<0.05, ##p<0.01, ###p<0.001 white vs. brite, *p<0.05, **p<0.01, ***p<0.001 no insulin vs. insulin stimulated.

So far, our data indicate that compromised insulin signaling does not interfere with the browning potential of white adipocytes and WAT. These findings prompted us to also address the question whether brite adipocytes, once recruited, differ from white fat cells in terms of insulin sensitivity. Browning is thought to improve the metabolic status by increasing glucose uptake from the circulation. However, it has not been fully understood so far whether this can be attributed to a simple increase in number of metabolically highly active brite adipocytes or if brite cells per se respond stronger to insulin stimulation than white cells. We therefore differentiated primary mouse adipocytes for 8 days with and without cPGI_2_ in the medium as described above. Efficient browning was verified by analyzing marker gene expression (Fig. S3A). Subsequently, cells were treated with specific doses (0–100 nM) of insulin for 20 minutes in the presence of a radioactively labeled non-metabolizable glucose derivative (^3^H-2-deoxyglucose, 3H-2DOG). Uptake of glucose was stimulated by insulin in a dose-dependent manner. The total amounts of incorporated 3H-2DOG upon insulin stimulation were substantially elevated in cells that underwent the brite compared to the white differentiation protocol. This was true in the basal state as well as upon insulin stimulation (Fig. 3A). To address the question if the two cell types differ in their reaction to the insulin stimulus, we calculated the relative increase in insulin-stimulated 3H-2DOG uptake by normalizing basal uptake to 1. Remarkably, the fold increase in glucose incorporation in response to any administered dose of insulin did not significantly differ between white and brite cells (Fig. 3B), indicating that both cell types responded to insulin stimulation in a similar manner.

In order to test the relevance of our findings in vivo, we induced browning in C57Bl6 mice by CL-administration employing subcutaneous osmotic pumps as described above. Following brite induction, CL-treated animals gained slightly but significantly less weight and displayed a loss of fat mass and white adipose tissue weights after 10 days compared to control animals, indicative of an improved metabolic status (Fig. S3B–D). This assumption was supported by the fact that basal serum glucose levels were lowered upon CL-treatment under random fed conditions (Fig. S3E). We next performed an intraperitoneal insulin tolerance test (ITT) to assess insulin response in CL and vehicle-treated animals. The generally lower serum glucose concentration of CL-treated animals was maintained throughout the ITT (Fig. 3C). However, considering the insulin effect in terms of relative decrease of blood glucose levels by normalization to the no-insulin group, we could not detect any significant difference between the CL- and the vehicle treated group (Fig. 3D). We next measured 3H-2DOG uptake into different tissues in CL-treated and control animals, with and without i.p. insulin injection. Whilst the relative enrichment of the tracer in blood serum was comparable in all 4 groups, indicating that any differences in total blood glucose did not bias the data by diluting the tracer (Fig. S3F), the uptake rate of the tracer into iWAT, aWAT and heart was augmented by insulin (Fig. 3E). Additionally, basal and insulin stimulated uptake of 3H-2DOG into WAT was markedly enhanced by CL-induced browning (Fig. 3E). The overall contribution of each depot to systemic glucose utilization per mouse was calculated from the respective uptake rate and weight of each tissue. In the white state, iWAT and aWAT roughly utilize only a third of the amount of glucose taken up by BAT, despite its higher abundance. In the brite state, the amount of whole-body glucose uptake attributable to WAT increases to the level contributed by the heart, but not to the level of BAT-associated glucose consumption in the basal, and almost to the same extent as BAT in the insulin stimulated state (Fig. S3G). Notably, while total 3H-2DOG uptake into WAT was substantially elevated upon browning, the relative fold increase glucose uptake as an effect of insulin stimulation did not differ between the two groups (Fig. 3F). In summary, our data show that brite recruitment in WAT increases systemic clearance of glucose from the bloodstream. However, the effect could not be attributed to improved insulin sensitivity of adipose tissue after browning.

### Browning of white adipose tissue induces GLUT-1 expression

**Figure 4:**
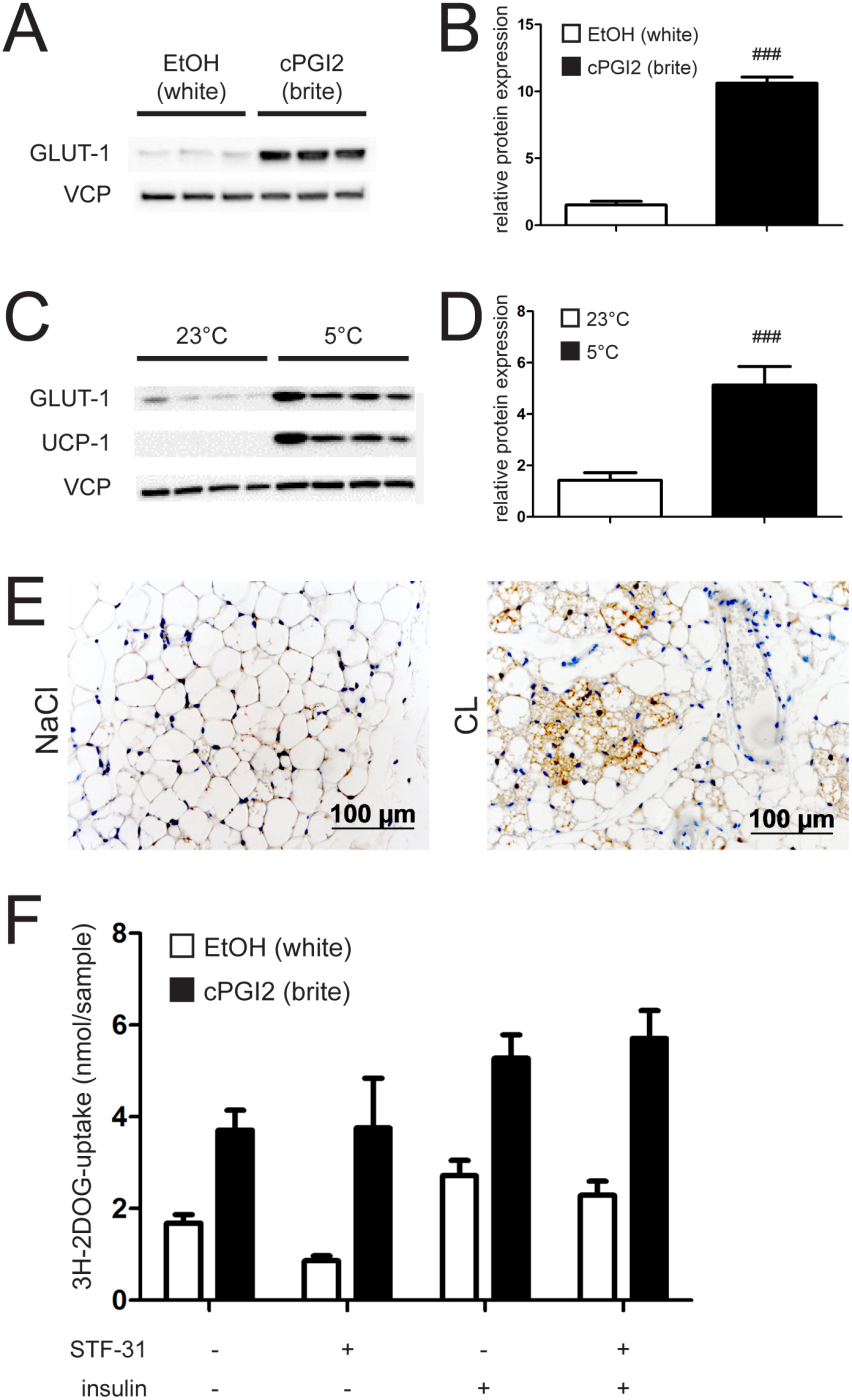
Browning induces GLUT-1 protein expression. (**A**) Representative immunoblot and (**B**) imageJ quantification of GLUT-1 from primary inguinal white adipose tissue (iWAT) precursor cells. Cells were differentiated into white (EtOH treated) or brite (cPGI_2_ treated) adipocytes for 8 days. (**C**) Representative immunoblot and (**D**) imageJ quantification of GLUT-1 from primary inguinal white adipose tissue (iWAT) of mice housed at 23°C and 5°C respectively for 10 days. (**E**) GLUT-1 stained iWAT slices of mice fed LFD and implanted with NaCl or CL s.c. pumps. (**F**) ^3^H-2-deoxy-D-glucose (3H-2DOG) uptake by primary inguinal white adipose tissue (iWAT) precursor cells. Cells were differentiated into white (EtOH treated) or brite (cPGI_2_ treated) adipocytes for 8 days, ± GLUT-1 inhibitor STF-31 for the last 2 days and stimulated with 20 nM Insulin for 20 min. All values are expressed as means ± SEM, n = 7 for in vivo experiments and 10–12 animals for cell isolation.

We next aimed to identify the insulin-independent mechanism underlying elevated 3H-2DOG uptake of brite adipose tissue. Since we found no significant upregulation of genes involved in glycolysis (data not shown), we hypothesized that insulin-independent glucose transporters might be responsible. To this end we analyzed expression of GLUT-1 in adipocytes differentiated with and without cPGI_2_. Glut-1 mRNA was slightly but significantly increased upon browning (Fig. S4A). Notably, we observed a substantial upregulation of GLUT-1 protein levels in treated cells (Fig. 4A, B).

To address the physiological relevance of GLUT-1 induction, we analyzed its expression in inguinal white fat depots of mice kept at 23°C or at 5°C for 10 days. Induction of the thermogenic program is illustrated by marked elevation of UCP-1 protein levels upon cold exposure (Fig. 4C). In accordance with in vitro results, housing at 5°C resulted in a robust induction of GLUT-1 in inguinal white adipose tissue after 10 days (Fig. 4C, D).

To validate this observation in an independent in vivo setting, we tested GLUT-1 induction upon browning induced by chronical CL treatment. Whereas Glut-1 mRNA levels were not substantially affected by CL (Fig. S4B), immunohistochemical analysis revealed that multilocular adipocytes arising upon CL-induced browning were GLUT-1 positive, whereas white cells displayed hardly any detectable GLUT-1 staining (Fig. 4E).

To demonstrate that the observed effect is insulin-independent, we probed for GLUT-1 induction in the pathological setting of insulin deficiency. Consistent with functional browning in the STZ mouse model (Fig. 2A), we found GLUT-1 protein to be significantly elevated upon CL administration in inguinal WAT of STZ-treated mice (Fig. S4C, D).

To functionally validate the impact of GLUT-1 on browning-stimulated glucose uptake, we employed the specific inhibitor STF 31 to block GLUT-1-dependent 3H-2DOG uptake by adipocytes. GLUT-1 inhibition by STF 31 significantly decreased basal glucose uptake (Fig. 4F). Expectedly, the inhibition of 3H-2DOG could be overcome by insulin treatment. Notably, also browning almost fully rescued the decrease in 3H-2DOG uptake by GLUT-1 inhibition (Fig. 4F), indicating that compensatory mechanisms control elevated glucose uptake during browning in the absence of GLUT-1 activity.

In summary, our data indicate that browning is accompanied by augmentation of GLUT-1 expression, thereby increasing glucose uptake into adipose tissue in an insulin-independent manner. Nevertheless, given that browning was able to compensate for the impaired glucose uptake caused by specific GLUT-1 inhibition, GLUT-1 induction only partially explains the enhanced glucose uptake of brite compared to white adipocytes.

## Discussion

The traditional concept of treating obesity and its co-morbidities by uncoupling the respiratory chain from ATP synthesis [40] re-emerged in recent years based on the unexpected discovery of active BAT depots in adult humans as well as the identification of brite adipocytes [3]–[5], [9], [10], [14], [41]. Several agents that induce or promote browning of preadipocytes or brite recruitment in WAT have been discovered, including sympathomimetics, prostaglandins, PPARγ activators, FGF21, natriuretic peptides and irisin [22], [36], [41]–[44].

There is clear evidence that obese, insulin resistant subjects display reduced brown fat activity [45], which might be primarily due to impaired brite recruitment [14], [16]–[19]. It is still not known though, whether aberrations in insulin signaling in obesity are cause or consequence of an impaired browning process or both. Hence, disturbances of the insulin signaling pathway might blunt brite adipocyte recruitment and/or activity, which would then engender a vicious cycle and further aggravate the metabolic status of affected patients. The results presented here indicate that absence of insulin signaling indeed affects differentiation of preadipocytes as well as the basal thermogenic state of white adipose tissue in vivo. This was not surprising, since it has been shown previously that insulin is crucial for the development of adipose tissues as well as thermogenesis in classical BAT [35]. Unexpectedly, we were able to observe effective browning upon β_3_-adrenergic stimulation by CL treatment in both a state of insulin deficit and a state of insulin resistance. Our findings thus support the idea that acute induction of browning per se is not insulin dependent and that activation of β_3_-adrenergic receptors is sufficient in this respect. Notably, our results are in line with a previous study on classical BAT, another thermogenically active adipose tissue, which reported that in this tissue thermogenesis induction is not strictly dependent on insulin/IGF-1 signaling [25].

Rodents are protected from obesity, hyperglycemia and insulin resistance when displaying high BAT activity [9]. Glitazones, which are clinically approved insulin sensitizers, increase BAT activity and induce browning, further suggesting that insulin responsiveness and browning are connected to a certain degree [41]. Congruently, we observed an improved systemic glucose clearance upon white adipose tissue browning in mice. In accordance with higher basal glucose utilization also the total amount of glucose cleared from the circulation specifically by WAT was generally higher in the brite state, and taking into account the higher abundance of WAT in terms of tissue mass it was found to be capable of clearing almost as much glucose from the system as BAT. Surprisingly, our approach revealed that brite adipose cells and tissue per se were not more insulin sensitive than white cells as indicated by the observation that relative increase of glucose uptake by insulin-stimulation was unaltered. Interestingly, a recent study comparing BAT activity in metabolically healthy young male human subjects, reported that there was no correlation of BAT activity and systemic insulin sensitivity as determined by FDG-PET/CT and hyperinsulinemic-euglycemic clamp, respectively [46]. Moreover, the recognized susceptibility of South-Asians to develop type 2 diabetes [47] could not be attributed to lower levels of active BAT [46]. It should be noted though that a more recent study raised the point that insulin sensitivity was improved in a cohort of five men upon BAT recruitment through cold acclimation [48]. Finally, in vitro studies provide evidence that β_3_-adrenergic stimulation of brown adipocytes triggers glucose uptake in an insulin-independent manner [49].

We interpret our findings in brite cells and tissue in a way that the decrease of blood glucose upon CL-induced browning is most probably caused by a generally higher, but insulin independent, glucose influx. It has been shown in skeletal muscle that exercise induces GLUT-4-driven glucose uptake independent from insulin [50]. However, we did not observe any changes in Glut-4 mRNA in adipose tissues upon brite recruitment (data not shown). The question remaining is what drives the massive glucose uptake of brite adipose tissue if not insulin? It has been shown in humans that two different mechanisms exist to boost glucose uptake of BAT, namely an insulin-dependent and an independent path, the latter relies on increased perfusion and might be exclusively relevant in cold activation [7]. A high perfusion rate is thus a critical determinant of BAT activity. Interestingly, cold exposure induces vascular endothelial growth factor (VEGF) expression in adipose tissue independently of hypoxia and tissue-specific overexpression of VEGF increases energy expenditure and improves whole-body insulin sensitivity and glucose tolerance in mice [51], [52]. In congruence with that, mice haploinsufficient for the retinoblastoma protein gene display improved glucose tolerance under an obesogenic diet which goes along with increased body core temperature and remodeling of WAT vascularization [53].

VEGF overexpression in adipose tissue is accompanied by WAT browning [54]. However, we could not detect an induction of VEGF during differentiation of adipocytes in the presence of cPGI_2_ (data not shown). We therefore concluded that remodeling of the vasculature is crucial for brite recruitment but not for its function per se. Also, induction of vascularization cannot explain the cell autonomous increase of glucose uptake that we have observed in brite adipocytes.

The GLUT-1 glucose transporter is expressed in several tissues and ensures insulin-independent glucose supply. Yet, it apparently plays a minor role in white adipose tissue [55]. Strikingly, classical brown adipocytes express high levels of GLUT-1 during differentiation and β_3_-adrenoreceptor agonism stimulates GLUT-1 expression and plasma membrane translocation in mature brown adipocytes [49]. Accordingly, we were able to show for the first time a substantial increase in GLUT-1 expression upon browning in adipocytes and in inguinal WAT. Thus, our results support the idea that browning at least partly stimulates glucose uptake into adipocytes by inducing the expression of an insulin-independent glucose transporter. The upstream cascade leading to GLUT-1 upregulation yet needs to be elucidated. It has been reported previously that Glut-1 transcription was activated by FGF21 [56]. Accordingly, we found Fgf21 mRNA to be induced twofold in adipocytes that underwent a browning protocol compared to those that underwent white differentiation (data not shown). It should be noted though that we observed full browning accompanied by elevation of GLUT-1 levels also in mice fed a HFD (data not shown) which are reported to be FGF21 resistant [56]. Thus, an alternative, yet unknown, pathway inducing GLUT-1 expression is most probably relevant during browning. As browning of adipocytes did overcome the inhibitory effect on 3H-2DOG uptake of a GLUT-1 blocker, GLUT-1 induction alone might only partially explain the increased glucose uptake and compensatory mechanisms might exist during browning. It appears likely that a multidimensional network, involving glucose uptake and turnover pathways, regulates sugar influx into brite adipocytes and that the effect of single components of this network in vivo might be overlaid by the increased amount of metabolically active cells.

## Conclusions

The beneficial effects of a higher BAT activity and brite recruitment on total energy expenditure are undisputed. Our data prove for the first time that recruitable brite adipocytes residing within white depots have the potential to decrease circulating glucose levels even in an insulin-resistant state by circumventing insulin signaling, which could be explained at least in part by induction of the glucose transporter GLUT-1. While it does not seem that impaired insulin sensitivity that can result from obesity impairs the capacity of WAT browning, it is well possible that innate variability in the recruitment of brite adipocytes might lower the capability of an individual to utilize glucose and thus render the subject more prone to obesity and insulin resistance.

Although induction of brite recruitment alone does thus not directly cure insulin resistance and type 2 diabetes, it is a promising approach to improve the disease state, since it has the potential to enduringly ameliorate hyperglycemia through a substantial increase of basal glucose uptake. Several factors that induce browning have been identified in the recent years, which should be further pursued regarding their therapeutic potential in humans in order to combat the “diabesity” pandemic.

## Supporting Information

Figure S1 **Insulin is required for proper adipocyte differentiation. (A)** Representative immunoblot of pAkt and total Akt from primary adipocyte precursor cells grown to 80% confluency and treated with the indicated doses of insulin and **(B)** quantification of the same blot with ImageJ. **(C)** mRNA expression of PGC1α and PPARγ in primary inguinal white adipose tissue (iWAT) precursor cells differentiated into white (EtOH treated) or brite (cPGI_2_ treated) adipocytes for 8 days with insulin present in the differentiation medium for the indicated timepoints (n = 3). All values in bar graphs are expressed as means ± SEM, #p<0.05, ##p<0.01, ###p<0.001 white (EtOH treated) vs. brite (cPGI_2_ treated) cells, *p<0.05, **p<0.01, ***p<0.001 normal conditions vs. insulin deprived conditions.
(TIF)

Figure S2 **Metabolic characterization of mouse models of hypo- and hyperinsulinemia. (A)** Random fed blood glucose levels and **(B)** serum insulin levels of streptozotocin (STZ, 60 µg/day/g bw) injected and control (upper panels) or 12 weeks low fat diet (LFD) or high fat diet (HFD) fed (lower panels) mice (n = 7–10). **(C)** Body weight over time and **(D)** inguinal and abdominal white or brown adipose tissue (iWAT, aWAT and BAT, respectively) weights at time of sacrifice of STZ injected and control (upper panels) or LFD and HFD fed (lower panels) mice (n = 7–10). **(E)** mRNA expression of Ucp-1, Cidea, cytochrome c oxidase subunit VIIa 1 (Cox7a1), cytochrome c oxidase subunit VIIIb (Cox8b) in iWAT (upper panel) and aWAT (lower panel) of mice either injected intraperitoneally (i.p.) or implanted with subcutaneous (s.c.) osmotic pumps, administering CL316,243 (CL) at a dose of 1 µg/g/day or control (NaCl) for 10 days (n = 3–5). **(F)** Tissue weights of iWAT (upper panel) and aWAT (lower panel) of i.p. or s.c. treated mice receiving CL or NaCl (n = 3–5). **(G)** ECHO-MRI body composition analysis, change in fat mass in STZ injected and control (upper panel) or LFD and HFD fed (lower panel) mice during the 10 days of CL or control treatment by s.c. pumps (n = 7–10). **(H)** Tissue weights of iWAT, aWAT, BAT, liver and gastrocnemius skeletal muscle (GC) of STZ injected and control (upper panel) or LFD and HFD fed (lower panel) mice implanted with CL or NaCl loaded s.c. pumps for 10 days (n = 7–10). **(I)** mRNA expression of UCP-1, CIDEA, CPT-1β, COX7a1, COX8b in aWAT of STZ injected (upper panel) and control mice or of LFD or HFD fed (lower panel) mice implanted with CL or NaCl loaded s.c. pumps for 10 days (n = 7–10). **(J)** UCP-1 stained aWAT slices of mice implanted with NaCl or CL s.c. pumps. Animals were fed a LFD or a HFD for 12 weeks. All values in bar graphs are expressed as means ± SEM, n = 7–10, #p<0.05, ##p<0.01, ###p<0.001 NaCl vs. CL, *p<0.05, **p<0.01, ***p<0.001 control vs. STZ and LFD vs HFD treated animals and i.p. vs. s.c. drug administration, respectively.
(TIF)

Figure S3 **Effects of browning in** **vitro and in** **vivo.** **(A)** mRNA expression of UCP-1, CIDEA, COX7a1, COX8b, CPT-1β, FABP4 and RETN in primary inguinal white adipose tissue (iWAT) precursor cells differentiated into white (EtOH treated) or brite (cPGI_2_ treated) adipocytes or in primary brown adipose tissue (BAT) precursor cells, all differentiated for 8 days. Values are shown as relative to expression in white adipocytes, except for Ucp-1 which was not detectable in white adipocytes and is expressed as relative to levels in brite adipocytes. **(B)** Change in body weight and **(C)** fat mass during 10 days of treatment in mice implanted with subcutaneous (s.c.) osmotic pumps administering CL316,243 (CL) at a dose of 1 µg/g/day or control (NaCl). **(D)** Inguinal and abdominal white adipose tissue (iWAT, aWAT) weights at time of sacrifice. **(E)** Basal blood glucose values upon CL or control treatment by s.c. pumps. **(F)** Relative enrichment of the ^3^H-2-deoxy-D-glucose (^3^H-2DOG) tracer in blood of mice implanted with NaCl or CL loaded s.c. pumps. Mice were injected with vehicle or 0.5 U/kg bw insulin over the 45 minutes course of the experiment. **(G)** Total ^3^H-2DOG uptake by each tissue of the mice shown in (F), calculated from the ^3^H-2DOG uptake and weight of each tissue. B–G: All values are expressed as means ± SEM, n = 6, #p<0.05, ##p<0.01, ###p<0.001 NaCl vs CL, *p<0.05, **p<0.01, ***p<0.001 basal vs insulin stimulation.
(TIF)

Figure S4 **Pharmacologically induced browning promotes GLUT-1 expression. (A)** mRNA expression of Glut-1 in primary inguinal white adipose tissue (iWAT) precursor cells. Cells were differentiated into white (EtOH treated) or brite (cPGI_2_ treated) adipocytes for 8 days. **(B)** mRNA expression of Glut-1 in primary inguinal white adipose tissue (iWAT) of mice housed at 23°C and 5°C respectively for 10 days. **(C)** Representative immunoblot and **(D)** imageJ quantification of GLUT-1 from primary inguinal white adipose tissue (iWAT) of STZ injected mice implanted with NaCl or CL loaded s.c. pumps. All values are expressed as means ± SEM, n = 7–10, #p<0.05, ##p<0.01, ###p<0.001 white vs. brite.
(TIF)
