# Supplementary figures and images for: Browning of White Adipose Tissue Uncouples Glucose Uptake from Insulin Signaling
Source: PLoS One. 2014 Oct 14;9(10):e110428. doi: 10.1371/journal.pone.0110428 (PMC4197027; doi:10.1371/journal.pone.0110428)

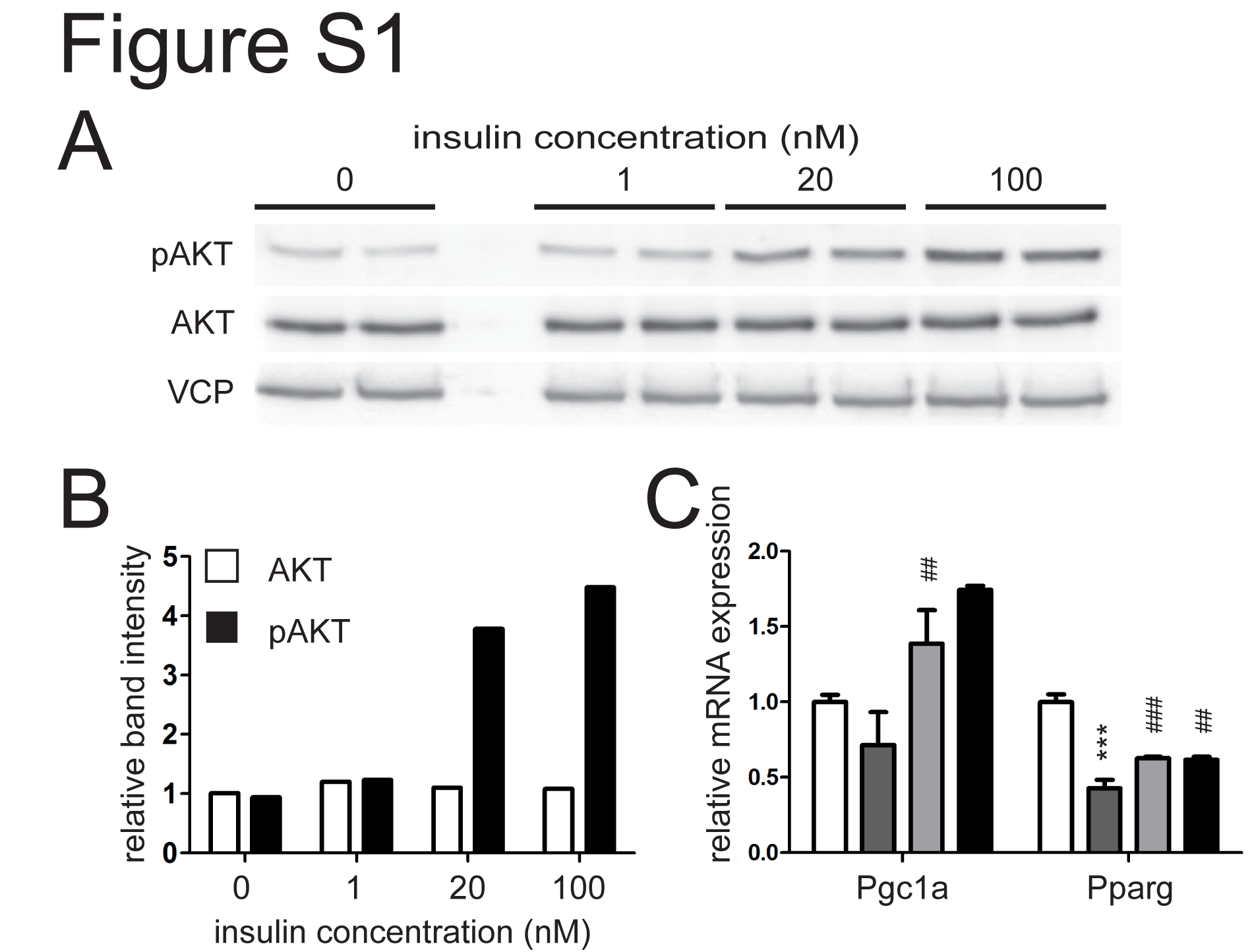

Supplement: Figure S1 — Insulin is required for proper adipocyte differentiation. (A) Representative immunoblot of pAkt and total Akt from primary adipocyte precursor cells grown to 80% confluency and treated with the indicated doses of insulin and (B) quantification of the same blot with ImageJ. (C) mRNA expression of PGC1α and PPARγ in primary inguinal white adipose tissue (iWAT) precursor cells differentiated into white (EtOH treated) or brite (cPGI2 treated) adipocytes for 8 days with insulin present in the differentiation medium for the indicated timepoints (n = 3). All values in bar graphs are expressed as means ± SEM, #p<0.05, ##p<0.01, ###p<0.001 white (EtOH treated) vs. brite (cPGI2 treated) cells, *p<0.05, **p<0.01, ***p<0.001 normal conditions vs. insulin deprived conditions. (TIF) [file pone.0110428.s001.tif]

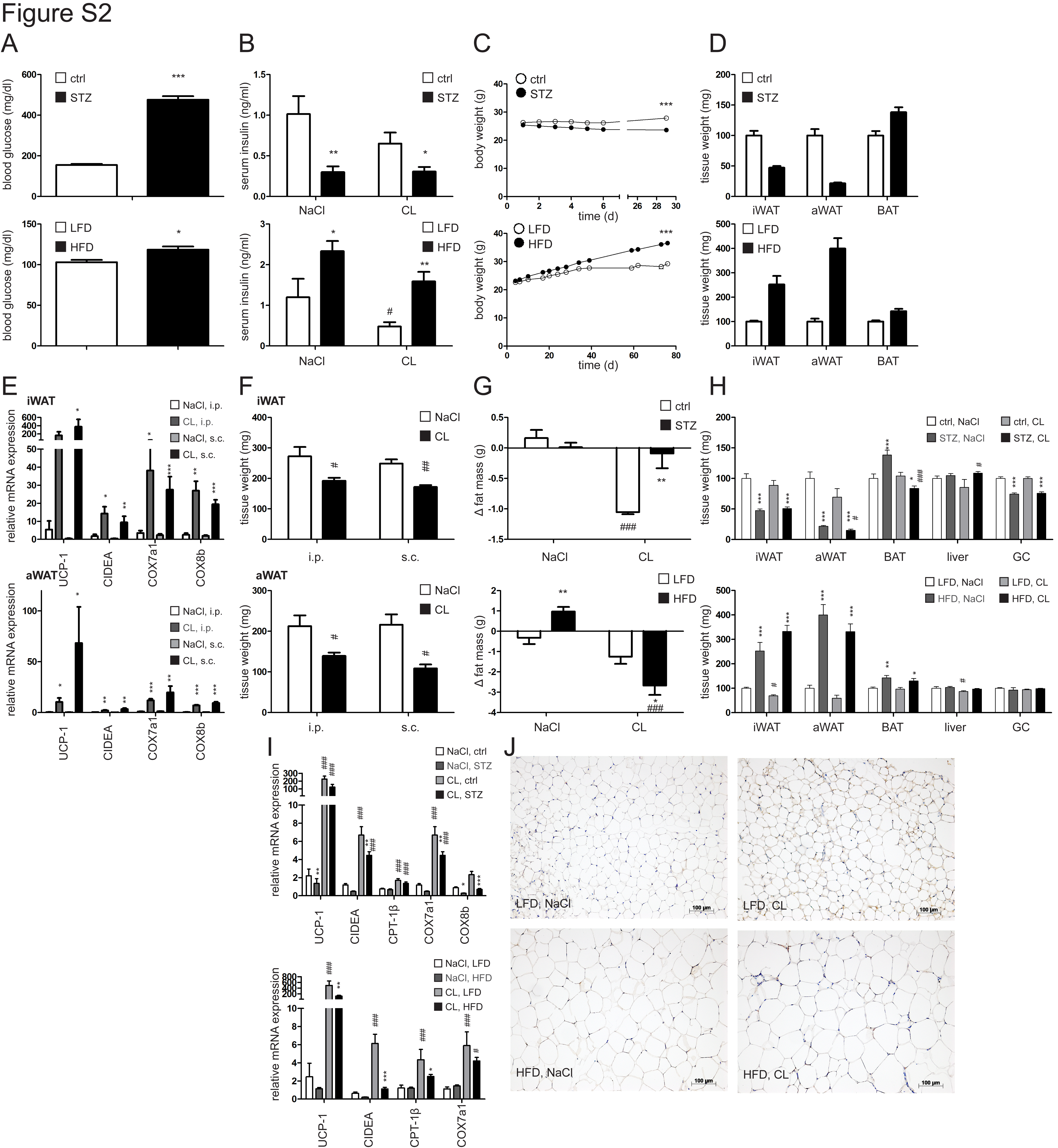

Supplement: Figure S2 — Metabolic characterization of mouse models of hypo- and hyperinsulinemia. (A) Random fed blood glucose levels and (B) serum insulin levels of streptozotocin (STZ, 60 µg/day/g bw) injected and control (upper panels) or 12 weeks low fat diet (LFD) or high fat diet (HFD) fed (lower panels) mice (n = 7–10). (C) Body weight over time and (D) inguinal and abdominal white or brown adipose tissue (iWAT, aWAT and BAT, respectively) weights at time of sacrifice of STZ injected and control (upper panels) or LFD and HFD fed (lower panels) mice (n = 7–10). (E) mRNA expression of Ucp-1, Cidea, cytochrome c oxidase subunit VIIa 1 (Cox7a1), cytochrome c oxidase subunit VIIIb (Cox8b) in iWAT (upper panel) and aWAT (lower panel) of mice either injected intraperitoneally (i.p.) or implanted with subcutaneous (s.c.) osmotic pumps, administering CL316,243 (CL) at a dose of 1 µg/g/day or control (NaCl) for 10 days (n = 3–5). (F) Tissue weights of iWAT (upper panel) and aWAT (lower panel) of i.p. or s.c. treated mice receiving CL or NaCl (n = 3–5). (G) ECHO-MRI body composition analysis, change in fat mass in STZ injected and control (upper panel) or LFD and HFD fed (lower panel) mice during the 10 days of CL or control treatment by s.c. pumps (n = 7–10). (H) Tissue weights of iWAT, aWAT, BAT, liver and gastrocnemius skeletal muscle (GC) of STZ injected and control (upper panel) or LFD and HFD fed (lower panel) mice implanted with CL or NaCl loaded s.c. pumps for 10 days (n = 7–10). (I) mRNA expression of UCP-1, CIDEA, CPT-1β, COX7a1, COX8b in aWAT of STZ injected (upper panel) and control mice or of LFD or HFD fed (lower panel) mice implanted with CL or NaCl loaded s.c. pumps for 10 days (n = 7–10). (J) UCP-1 stained aWAT slices of mice implanted with NaCl or CL s.c. pumps. Animals were fed a LFD or a HFD for 12 weeks. All values in bar graphs are expressed as means ± SEM, n = 7–10, #p<0.05, ##p<0.01, ###p<0.001 NaCl vs. CL, *p<0.05, **p<0.01, ***p<0.001 control vs. STZ and LFD vs HFD tr [file pone.0110428.s002.tif]

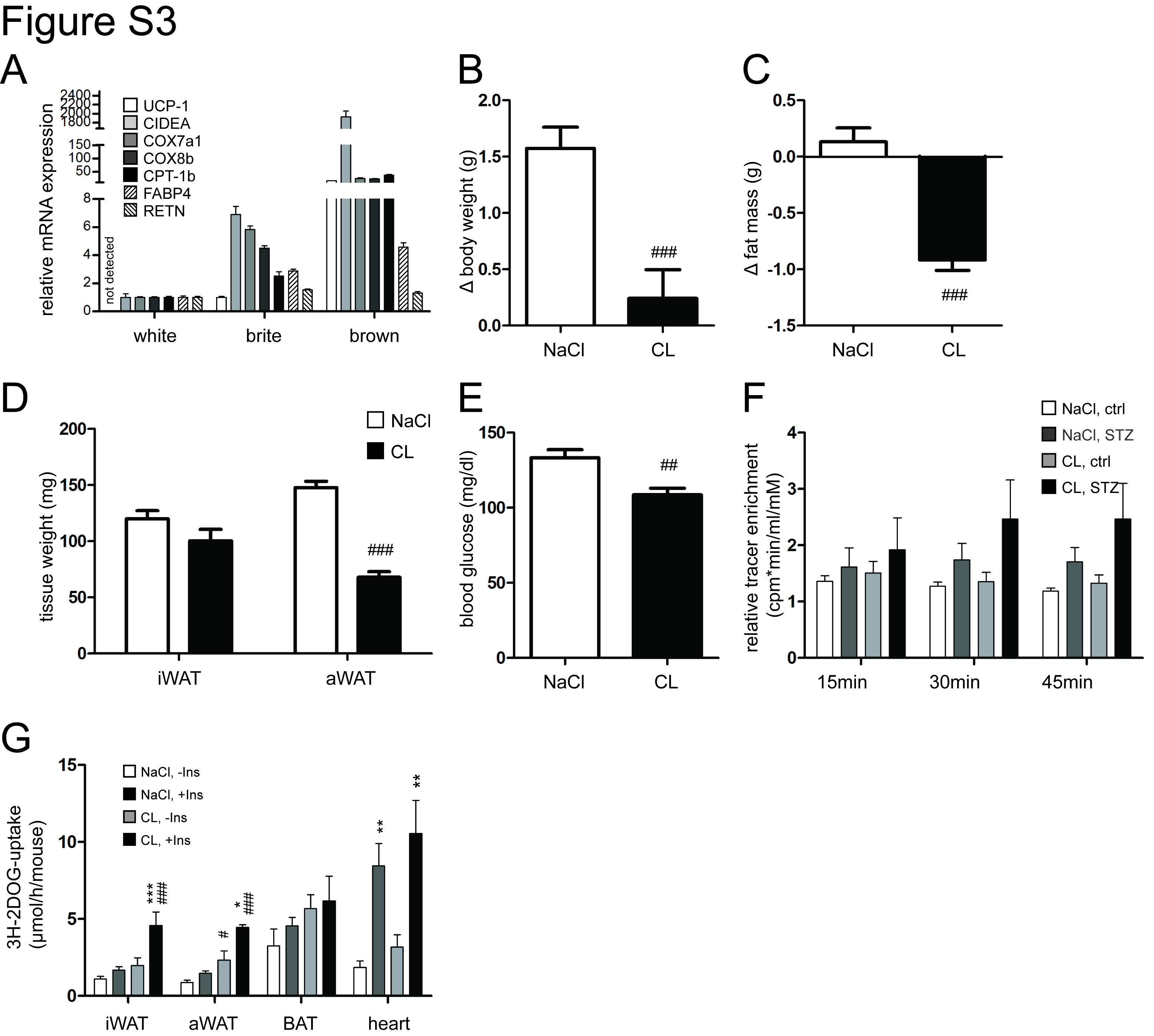

Supplement: Figure S3 — Effects of browning in vitro and in vivo. (A) mRNA expression of UCP-1, CIDEA, COX7a1, COX8b, CPT-1β, FABP4 and RETN in primary inguinal white adipose tissue (iWAT) precursor cells differentiated into white (EtOH treated) or brite (cPGI2 treated) adipocytes or in primary brown adipose tissue (BAT) precursor cells, all differentiated for 8 days. Values are shown as relative to expression in white adipocytes, except for Ucp-1 which was not detectable in white adipocytes and is expressed as relative to levels in brite adipocytes. (B) Change in body weight and (C) fat mass during 10 days of treatment in mice implanted with subcutaneous (s.c.) osmotic pumps administering CL316,243 (CL) at a dose of 1 µg/g/day or control (NaCl). (D) Inguinal and abdominal white adipose tissue (iWAT, aWAT) weights at time of sacrifice. (E) Basal blood glucose values upon CL or control treatment by s.c. pumps. (F) Relative enrichment of the 3H-2-deoxy-D-glucose (3H-2DOG) tracer in blood of mice implanted with NaCl or CL loaded s.c. pumps. Mice were injected with vehicle or 0.5 U/kg bw insulin over the 45 minutes course of the experiment. (G) Total 3H-2DOG uptake by each tissue of the mice shown in (F), calculated from the 3H-2DOG uptake and weight of each tissue. B–G: All values are expressed as means ± SEM, n = 6, #p<0.05, ##p<0.01, ###p<0.001 NaCl vs CL, *p<0.05, **p<0.01, ***p<0.001 basal vs insulin stimulation. (TIF) [file pone.0110428.s003.tif]

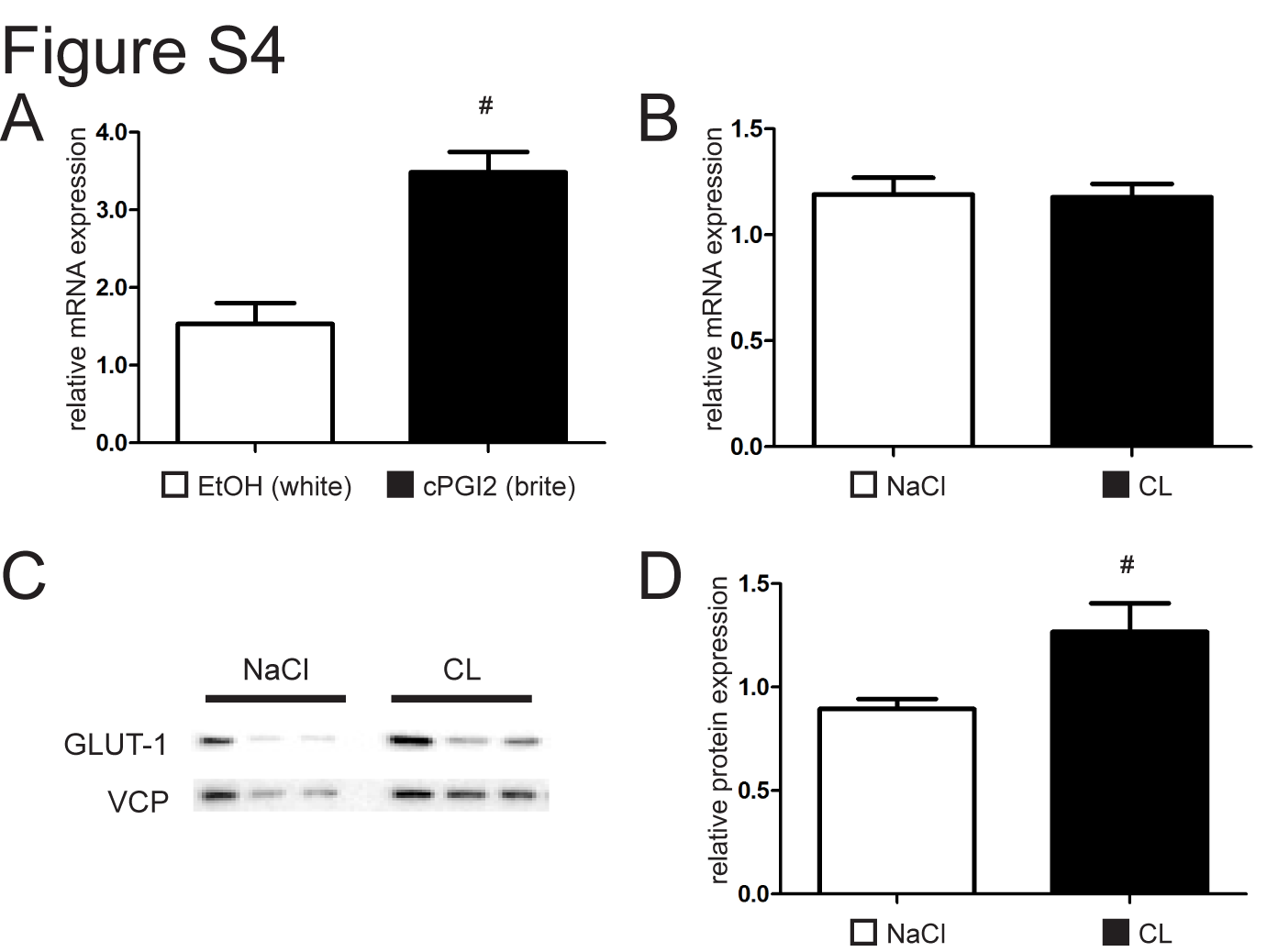

Supplement: Figure S4 — Pharmacologically induced browning promotes GLUT-1 expression. (A) mRNA expression of Glut-1 in primary inguinal white adipose tissue (iWAT) precursor cells. Cells were differentiated into white (EtOH treated) or brite (cPGI2 treated) adipocytes for 8 days. (B) mRNA expression of Glut-1 in primary inguinal white adipose tissue (iWAT) of mice housed at 23°C and 5°C respectively for 10 days. (C) Representative immunoblot and (D) imageJ quantification of GLUT-1 from primary inguinal white adipose tissue (iWAT) of STZ injected mice implanted with NaCl or CL loaded s.c. pumps. All values are expressed as means ± SEM, n = 7–10, #p<0.05, ##p<0.01, ###p<0.001 white vs. brite. (TIF) [file pone.0110428.s004.tif]
